# Outcomes of invasive mechanical ventilation in critically ill patients with interstitial lung disease: protocol for a systematic review and meta-analysis

**DOI:** 10.1136/bmjopen-2026-118165

**Published:** 2026-06-08

**Authors:** Novis Kola, Robert Parker, Angela Hall, Lisa G Spencer, Sophie V Fletcher, Ingeborg D Welters, Brian W Johnston

**Affiliations:** 1 Intensive Care Unit, Royal Liverpool University Hospital, Liverpool, UK; 2 Intensive Care Unit, Aintree University Hospital, Liverpool, UK; 3 Faculty of Health and Life Sciences, University of Liverpool, Liverpool, UK; 4 Liverpool Centre for Cardiovascular Science, University of Liverpool, Liverpool, UK; 5 Thoracic Medicine, Aintree University Hospitals NHS Foundation Trust, Liverpool, UK; 6 Respiratory Medicine, University Hospital Southampton NHS Foundation Trust, Southampton, UK; 7 School of Clinical and Experimental Sciences, University of Southampton, Southampton, UK; 8 Institute of Life Course and Medical Sciences, University of Liverpool, Liverpool, UK; 9 Liverpool Centre for Cardiovascular Sciences, University of Liverpool Faculty of Health and Life Sciences, Liverpool, UK

**Keywords:** Mortality, Interstitial lung disease, INTENSIVE & CRITICAL CARE, Ventilators, Mechanical, RESPIRATORY MEDICINE (see Thoracic Medicine)

## Abstract

**Abstract:**

**Introduction:**

Interstitial lung diseases (ILDs) represent a heterogeneous group of disorders, which have in common persistent inflammation and/or pulmonary fibrosis, involving mainly but not exclusively the interstitium. This results in restrictive ventilatory physiology and limited respiratory reserve. Patients with ILD can have frequent exacerbations of their disease, with subsequent acute respiratory failure that may require admission to the intensive care unit (ICU). The diagnosis and management of ILD in the ICU presents unique challenges due to the paucity of evidence supporting survival benefits of organ support in this cohort of patients.

**Methods and analysis:**

This systematic review will be reported in accordance with the Preferred Reporting Items for Systematic Reviews and Meta-Analyses (PRISMA) statement, and the protocol will follow the Preferred Reporting Items for Systematic Review and Meta-Analysis Protocols (PRISMA-P) guideline. MEDLINE, Embase, Emcare and CENTRAL will be searched for studies published from inception to 2026, involving adult patients with ILD requiring invasive mechanical ventilation (IMV), with or without comparison to non-invasive respiratory support such as high-flow oxygen, non-invasive ventilation (NIV), continuous positive airway pressure or bilevel positive airway pressure. Eligible studies will include randomised controlled trials and observational studies (cohort and case–control) in adults with ILD and acute respiratory failure requiring IMV in the intensive care setting. Case series with fewer than 10 patients, non-human or in vitro studies and studies involving perioperative lung transplant or lung cancer as the primary diagnosis will be excluded. The primary outcomes assessed will be in-hospital and 1-year mortality, and secondary outcomes will include ventilator-free days, ICU and hospital length of stay, NIV failure, reintubation and postdischarge respiratory outcomes where available. Where feasible, meta-analysis will be conducted using a random-effects model. Heterogeneity will be assessed using the I² statistic. Prespecified subgroup analyses will be performed, including ILD subtype (eg, idiopathic pulmonary fibrosis (IPF) vs non-IPF), presence of pulmonary hypertension, timing of IMV initiation (early vs late), baseline lung function (forced vital capacity ≥50% vs <50%), ventilation strategy (lung protective vs non-specified) and geographical region (eg, Europe, North America, Asia). Sensitivity analyses will be conducted to evaluate robustness of results. Risk of bias will be assessed using the Cochrane Risk of Bias 2 tool (RoB 2) for randomised studies and the Newcastle-Ottawa Scale for non-randomised studies. The quality of evidence will be assessed by using the Grading of Recommendations Assessment, Development and Evaluation approach.

**Ethics and dissemination:**

This systematic review will be based on published data, and as such, no ethical approval is required. Findings from this study will be disseminated through peer-reviewed publications as well as presentations in healthcare-based settings.

**PROSPERO registration number:**

CRD420251265836.

**STRENGTHS AND LIMITATIONS OF THIS STUDY**

- The protocol is reported in accordance with the Preferred Reporting Items for Systematic Review and Meta-Analysis Protocols (PRISMA-P) guideline to enhance transparency and completeness of reporting.
- A comprehensive search strategy across multiple databases will minimise the risk of missing relevant studies.
- Planned meta-analyses will allow pooled outcome estimates and assessment of statistical heterogeneity where appropriate.
- Inclusion of observation studies may increase susceptibility to bias and residual confounding.
- Clinical and methodological heterogeneity across studies may limit comparability and synthesis of findings.

## Introduction

 Interstitial lung diseases (ILDs) encompass a wide group of diffuse parenchymal lung diseases that are classified together due to similarities in various aspects of their clinical, radiological, physiological and pathological manifestations. The various subgroups of ILDs include idiopathic ILDs, autoimmune-related ILDs, exposure-related ILDs (including iatrogenic) and ILDs with cysts or airspace filling, sarcoidosis and orphan diseases. Their hallmark is the presence of inflammation, fibrosis or a combination of both, involving the pulmonary interstitium in the early stages and in a variety of cases progressively extending to the alveoli and airways. This leads to disruption of the architecture of the lungs, reduced lung function and impaired gas exchange with subsequent symptoms of dyspnoea and fatigue. Despite common shared characteristics, there is significant variability in the clinical course of the different ILDs from completely reversible to self-limiting to progressive, irreversible and often fatal, despite optimal management. This distinction is particularly evident when considering ILDs with a non-specific interstitial pneumonia pattern, which tend to reverse and stabilise with treatment, while in other ILDs almost a third of patients will ultimately manifest pulmonary fibrosis, regardless of the specific diagnosis. When overt progressive pulmonary fibrosis manifests, there is a significant increase in morbidity and mortality. This is particularly relevant in the case of idiopathic pulmonary fibrosis (IPF), which represents the most common fibrotic ILD but also applies to other fibrotic ILDs.^1^,^3^

Limited physiological respiratory reserve, associated with possible extrapulmonary complications of the disease including secondary pulmonary hypertension, increases the risk of severe acute respiratory failure during disease exacerbations. These episodes of exacerbation can lead to admission to hospital and in some cases admission to level 2 or 3 care settings.^4^,^7^

Despite significant developments on pathogenetic mechanisms and subsequent advances in the management of patients with ILDs, data from several observational studies suggest that when admitted to hospital due to acute disease exacerbation, in-hospital mortality, as well as mortality in the postdischarge period, remains high, particularly in patients with IPF.^8^,^10^ There is, however, concordance among different studies that there is a significant difference in mortality among patients affected by different ILDs, with considerably higher rates of mortality in patients with IPF as compared with those affected by non-IPF ILDs.^9^,^13^

In a more detailed review of this cohort of patients, the current management of their acute respiratory failure, and in particular severe acute respiratory failure, is heavily based on expert consensus. Robust evidence to support the care of patients with ILD admitted to intensive care unit (ICU) with acute respiratory failure, requiring progressively increasing supplemental oxygen to maintain adequate oxygenation, is lacking. Supplemental oxygenation is usually delivered in ICU through high-flow oxygen (HFO), non-invasive ventilation (NIV) and invasive mechanical ventilation (IMV)^4^ There is emerging evidence from various studies that both HFO and NIV might be a valid therapeutic alternative to IMV, allowing at least hypothetically to reduce the morbidity and mortality associated with IMV in these patients.^514^,^16^

The aim of this study is to provide a contemporary synthesis of the current available evidence of outcomes in patients with ILD and acute respiratory failure admitted to ICU requiring ventilatory support.

## Methods and analysis

### Protocol design and registration

The study protocol for this systematic review will be reported in accordance with the Preferred Reporting Items for Systematic Review and Meta-Analysis Protocols (PRISMA) guideline, and the systematic review will be reported in accordance with the Preferred Reporting Items for Systematic Reviews and Meta-Analyses (PRISMA) statement principles and the Meta-Analyses and Systematic Reviews of Observational Studies (MOOSE) guideline.^17^,^21^

The protocol has been registered with the International Prospective Register of Systematic Reviews (PROSPERO; CRD420251265836).

Initial planning for the systematic review started in November 2025, and after initial literature screening as well as refining of our search terms, formal search of the literature from inception to 2026 is being conducted. We aim to start our systematic review in April 2026 and complete it by September 2026.

### Eligibility criteria

#### The population

Randomised as well as observational studies of human adults aged >18 years with any form of ILD, requiring oxygen support therapy due to acute respiratory failure in intensive care settings, will be included. Level of support in ICU will be level 2 or level 3 as defined by the authors of the studies themselves.

Studies including non-human patients, in vitro setting, patients with lung cancer as the primary diagnosis, pregnant patients, perioperative lung transplant patients or case reports of less than 10 patients will be excluded (table 1).

**Table 1:** Inclusion and exclusion criteria

Inclusion criteria	Exclusion criteria
Human studies Adults aged over 18 years Randomised studies Observational studies Patient with acute respiratory failure and any form of interstitial lung disease Intensive care setting	Non-human studies In vitro studies Pregnant patients Lung cancer as primary diagnosis Perioperative lung transplant patients Case reports of less than 10 patients

#### Intervention and comparators

The intervention of interest in this review is IMV. IMV is defined as the provision of ventilatory support via an artificial airway, including both endotracheal intubation and tracheostomy. Comparators include other alternative ways of oxygen delivery such as HFO, continuous positive airway pressure and bilevel positive airway pressure in intensive care settings.

#### Outcomes

The primary outcomes that will be evaluated include in-hospital and 1-year mortality. The secondary outcomes that will be evaluated include ventilator-free days, ICU length of stay, hospital length of stay, NIV failure, need for reintubation and postdischarge respiratory outcomes where available.

NIV failure is defined as a requirement for transition from NIV to IMV due to clinical deterioration, which can present in the way of worsening hypoxaemia (PaO_2_/FiO_2_ ratio decline), persistent or worsening respiratory distress, hypercapnia with acidosis, haemodynamic instability or altered mental status, as defined by the authors of the included studies. We will report the definitions used for NIV failure in the specific studies.

Reintubation is defined as the requirement for repeat endotracheal intubation within 48 hours following planned extubation or tracheostomy decannulation. Where studies report alternative timeframes (eg, 72 hours or 7 days), these will be extracted and analysed separately where feasible. Study-specific definitions and indications for reintubation will be recorded.

Postdischarge respiratory outcomes are defined as any of the following: requirement for long-term oxygen therapy, decline in lung function, defined as ≥10% absolute decline in forced vital capacity (FVC), or ≥15% decline in diffusing capacity of the lungs for carbon monoxide, respiratory-related rehospitalisation or need for chronic ventilatory support.

If study-specific definitions differ from the prespecified ones above described, they will be recorded but no pooling will be undertaken, unless definitions are sufficiently homogeneous.

#### Information sources and search strategy

Current available literature will be searched in a systematic manner through four databases for studies published from inception until 2026: Medical Literature Analysis and Retrieval System Online (MEDLINE), Excerpta Medica Database (Embase), Emcare, all via OVID platform, and Cochrane Central Register of Controlled Trials (CENTRAL). The search strategy was developed with the help of a research librarian (AH). The following key concepts were considered relevant: ILD, IMV, critically ill patients and outcomes. A list of keywords will be used in all four databases to search for articles, with a title or abstract featuring at least one keyword from the following: ILDs, oxygen inhalation therapy, positive-pressure respiration, critical care and mortality.

Throughout the search strategy, Boolean operators (OR, AND), truncation (*) for various suffixes and quotation marks (“”) for phrases will be used.

The search will include literature available up to the date of the final search from inception, with no restrictions on publication language or geographical location. The full search strategy is available in table 2.

**Table 2:** Search strategy and databases searched

Database	Search strategy
MEDLINE/Emcare/Embase via OVID	exp Lung Diseases, Interstitial/ exp Idiopathic Pulmonary Fibrosis/ exp Idiopathic Interstitial Pneumonias/ (“interstitial lung disease*” or “ILD*” or pulmonary fibrosis* or “fibrotic lung disease*” or “interstitial pneumon*” or “idiopathic pulmonary fibrosis*” or “IPF”).kf,tw. 1 or 2 or 3 or 4 exp Positive-Pressure Respiration/ Respiration, Artificial/ Ventilators, Mechanical/ (“mechanical ventilation” or “invasive ventilation” or “invasive mechanical ventilation” or “IMV” or intubat*).kf,tw. 6 or 7 or 8 or 9 exp Oxygen Inhalation Therapy/ Noninvasive Ventilation/ (“High-Flow Nasal Cannula Therap*” or “oxygen therap*” or “supplement* oxygen” or “nasal cannula*” or “HFNC” or “high flow oxygen” or “non-invasive ventilation” or “NIV” or “CPAP” or “BiPAP”).kf,tw. 11 or 12 or 13 Intensive Care Units/ Critical Care/ Critical Illness/ (“intensive care unit*” or “ICU*” or “critical care unit*” or “CCU” or “Intensive therapy unit*” or “ITU” or “Intensive care” or “critical care” or “Intensive therap*” or “critical* ill*").kf,tw. 15 or 16 or 17 or 18 mortality/ or cause of death/ or fatal outcome/ or hospital mortality/ or survival rate/ (mortalit* or death* or surviv* or died or dying or dead).kf,tw. 20 or 21 5 and 10 and 14 and 19 and 22
CENTRAL	MeSH descriptor: [Lung Diseases, Interstitial] explode all trees MeSH descriptor: [Pulmonary Fibrosis] explode all trees MeSH descriptor: [Idiopathic Pulmonary Fibrosis] explode all trees 1 OR 2 OR 3 “interstitial lung disease” OR ILD* OR “diffuse parenchymal lung disease” OR DPLD* OR pulmonary fibrosis* OR fibrotic lung disease OR “idiopathic pulmonary fibrosis” OR IPF* OR “idiopathic interstitial pneumonia” OR IIP* 4 OR 5 MeSH descriptor: [Respiration, Artificial] explode all trees MeSH descriptor: [Positive-Pressure Respiration] explode all trees MeSH descriptor: [Oxygen Inhalation Therapy] explode all trees MeSH descriptor: [Noninvasive Ventilation] explode all trees 7 OR 8 OR 9 OR 10 ventilat* OR intubat* OR “mechanical ventilation” OR “noninvasive ventilation” OR NIV OR CPAP OR BiPAP OR “high flow nasal cannula” OR HFNC OR oxygen OR oxygen therap* OR supplemental oxygen OR respiratory support 11 OR 12 MeSH descriptor: [Critical Care] explode all trees MeSH descriptor: [Critical Illness] explode all trees MeSH descriptor: [Intensive Care Units] explode all trees 14 OR 15 OR 16 “intensive care” OR ICU OR intensive care unit* OR “critical care” OR “critical illness” OR CCU OR ITU 17 OR 18 MeSH descriptor: [Mortality] explode all trees MeSH descriptor: [Death] explode all trees MeSH descriptor: [Survival] explode all trees MeSH descriptor: [Fatal Outcome] explode all trees 20 OR 21 OR 22 OR 23 mortalit* OR death* OR died OR dying OR fatal* 24 OR 25 6 AND 13 AND 19 AND 26

For studies published in languages other than English, we will adopt two approaches. First, multilingual members of our team, specifically those proficient in Italian and Spanish, will review studies published in these languages. Second, for articles published in other languages, we will contact the study authors to request an English version. If an English version is available, the study will be included.

Grey literature (eg, conference abstracts, theses, unpublished reports and author collections) will be screened and included only if sufficient clinical detail is available and the study meets predefined inclusion and exclusion criteria. Sources lacking adequate data will be excluded, as conference abstracts and similar materials often provide incomplete or inconsistently reported information. In cases where data are available both as a conference abstract and a full peer-reviewed publication, only the published study will be included to avoid duplication and ensure completeness of reporting.

Overall, prioritising peer-reviewed studies supports the inclusion of high-quality, fully reported data, thereby improving the reproducibility and validity of the review. This approach is consistent with guidance from the Cochrane Handbook for Systematic Reviews of Interventions, which allows for the exclusion of grey literature when methodological rigour and completeness of outcomes are prioritised.

#### Study selection

Search results will be imported into a reference management software, and duplicate studies will be removed automatically. The software to be used will be Rayyan (Rayyan Systems, Cambridge, Massachusetts, USA).

Screening will be conducted in two stages by two independent reviewers (NK, BWJ), with any disagreement arising from this to be resolved by a third reviewer (RP). First-stage screening will include title and abstract screening to identify potentially relevant studies.

Second-stage screening will include full-text screening of the articles selected during the first phase based on predefined inclusion criteria. Eligible studies will be included in the systematic review and, where appropriate, in the meta-analysis.

The above-described selection process will be presented in the PRISMA flow diagram.

#### Data extraction

Data will be extracted into a Microsoft Excel (Microsoft, Redmond, Washington, USA) spreadsheet by two independent reviewers. The following information will be collected from the studies included: study characteristics, patient characteristics, modality of oxygen therapy support and outcomes. The full details of these are reported in table 3.

**Table 3:** Detailed data extraction of items

Category	Data items	Notes
Study characteristics	First author Year Country/region Study design Sample size Study period Data source Funding	
Patient characteristics	Age Sex distribution BMI ILD subtype APACHE II score Baseline FVC% predicted, DLCO% predicted Comorbidities (CVD, PH, COPD, asthma, CKD, T2DM)	
Intervention/comparator details	Timing (early vs late) IMV modality (ETT vs tracheostomy) IMV parameters: mode, TV, PEEP, FiO_2_%, PS, RespR, I/E ratio NIV settings: IPAP, EPAP, backup RespR CPAP settings: pressure, FiO_2_% HFO settings: FiO_2_%, flow	If reported.
Outcomes	Author-reported mortality outcomes Author-reported ventilator-free days Author-reported ICU and hospital LOS (days) Author-reported NIV failure Author-reported reintubation need Author-reported postdischarge respiratory outcomes	Outcomes will be assessed against prespecified definitions*; and when they differ, this will be clearly reported.

BMI is measured in kilograms per square metre; DLCO% predicted is the measured DLCO expressed as a percentage of the expected value for a healthy individual of the same age, sex, height and haemoglobin level; FiO_2_% is the percentage of oxygen in the gas mixture that a patient inhales; flow is measured in litres per minute; FVC% predicted is measured as per cent-predicted FVC in comparison with the expected value for a healthy individual of the same age, sex, height and ethnicity; pressure, PEEP, IPAP, EPAP and PS are measured in cmH2O; RespR is measured in breaths per minute; I/E is measured in the ratio of the duration of inspiration (I) to the duration of expiration (E) during mechanical ventilation in units of time; TV is measured in millilitres.

* See the Outcomes subsection under the Eligibility criteria section.

APACHE II, Acute Physiology and Chronic Health Evaluation II; BMI, body mass index; CKD, chronic kidney disease; COPD, chronic obstructive pulmonary disease; CPAP, continuous positive airway pressure; CVD, cardiovascular disease; DLCO, diffusing capacity of the lungs for carbon monoxide; EPAP, expired positive airway pressure; ETT, endotracheal tube; FiO_2_, fraction of inspired oxygen; FVC%, forced vital capacity predicted in percentage; HFO, high-flow oxygen; ICU, intensive care unit; I/E, inspiration to expiration ratio; ILD, interstitial lung disease; IMV, invasive mechanical ventilation; IPAP, inspired positive airway pressure; LOS, length of stay; NIV, non-invasive ventilation; PEEP, positive end-expiratory pressure; PH, pulmonary hypertension; PS, pressure support; RespR, respiratory rate; T2DM, type 2 diabetes mellitus; TV, tidal volume.

For missing or unclear data, we will contact the study authors and request for the data or clarification. If data remain unavailable, we will use the available case analysis, conduct sensitivity analyses to assess the impact of missing data and report the extent and nature of missing data transparently.

#### Data synthesis

Data synthesis will be conducted via R (V.4.5.2; R Foundation for Statistical Computing).

Data will be combined using a random-effects model for meta-analysis, as we expect substantial heterogeneity in the populations, interventions and settings across studies.

For continuous outcomes (eg, ventilator-free days, ICU length of stay), we will calculate the mean difference (MD) or standardised mean difference (SMD) where appropriate, depending on whether the outcome measures are consistent across studies. For binary outcomes (eg, in-hospital mortality, NIV failure, reintubation), we will calculate risk ratios (RR) or ORs, depending on the reporting method in the included studies.

The effect sizes will be pooled using a random-effects model to account for both within-study and between-study variability. This model assumes that the true effect size may differ between studies. A weighted average of effect sizes will be calculated, with larger studies (or studies with less variance) given more weight in the pooled estimate.

Given that ILD encompasses a heterogeneous group of conditions with differing pathological mechanisms and clinical courses, variation in outcomes following IMV is anticipated across ILD subtypes.

Despite this heterogeneity, all ILD subtypes will be pooled in the primary analysis. This approach is justified by the primary objective of the review, which is to estimate overall outcomes of IMV in patients with ILD as a broad clinical group. In practice, decisions regarding IMV are frequently made in acute settings where ILD subtype classification may be incomplete or unavailable, supporting the clinical relevance of a pooled estimate. Furthermore, pooling is expected to enhance statistical power given the likely small sample sizes within individual ILD subtypes.

We will assess heterogeneity across studies using the I² statistic. A value greater than 50% will indicate significant variability between studies, and subgroup analyses will be performed to explore potential sources of this heterogeneity (eg, ILD subtype, type of mechanical ventilation or geographical differences in treatment).

The χ^2^ test will be used to test for the statistical significance of heterogeneity.

Subgroup analyses will be conducted to explore the influence of study-level factors, where data are available.

The following subgroup analyses are planned (subject to data availability): ILD subtype (eg, IPF vs non-IPF); presence of pulmonary hypertension; timing of IMV initiation (early vs late); IMV modality baseline lung function (FVC ≥50% vs <50%); ventilation strategy (lung protective vs non-specified); and geographical region (eg, Europe, North America, Asia).

These analyses aim to explore potential sources of heterogeneity. Where sufficient studies are available, meta-regression may also be conducted.

Sensitivity analyses will be performed to evaluate the robustness of our results, particularly by excluding studies with high risk of bias or significant methodological differences. The results will be presented as forest plots, with individual study effect sizes and the overall pooled effect size shown.

For continuous outcomes, the pooled effect size will be reported as the MD or SMD; for binary outcomes, it will be reported as the RR or OR, with corresponding 95% CIs.

We will assess publication bias when at least 10 studies are included in a meta-analysis. This will be done using funnel plots which will be visually inspected for asymmetry and Egger’s regression test which will allow statistical evaluation of small study effects.

If there are insufficient data to allow meta-analysis for specific outcomes, a narrative synthesis will be provided.

#### Risk of bias assessment and quality of evidence

The risk of bias will be appraised using the Cochrane RoB 2 tool for randomised controlled trials and the Newcastle-Ottawa Scale (NOS) tool for observational studies.

The RoB 2 tool consists of bias domains, each assessed using signalling questions which lead to judgements about the risk of bias for the domain. The domains considered include the randomisation process, deviations from intended interventions, missing outcome data, measurements of the outcome and selection of the reported result. In addition to these, there is an overall bias section. For all of these, the risk of bias can be low, some concerns or high.^22^

The NOS allows an assessment of risk of bias in observational studies through evaluation of three domains on the studies: selection, comparability and outcome (cohort studies)/exposure (case–control studies), which then contain a total of eight items. A semiquantitative assessment of study quality is done through a star system, where each item is awarded one star, except for the item related to comparability that allows the assignment of two stars. The overall NOS ranges from zero to nine stars.^23^

The quality of evidence for each outcome will be assessed using the Grading of Recommendations Assessment, Development and Evaluation (GRADE) approach. GRADE provides a systematic and transparent framework for rating the confidence in effect estimates across studies for each critical outcome. Under GRADE, evidence derived from randomised controlled trials will initially be rated as high certainty, while evidence from observational studies will initially be rated as low certainty. The quality of evidence can be upgraded or downgraded considering several domains on the studies considered.^24 25^

Data will be assessed independently by at least two reviewers, with a process to resolve differences after discussion and revision from a third reviewer, if required. Additional information will be sought from study investigators if required information is unclear or unavailable in the study publications/reports.

## Discussion

This systematic review aims to synthesise evidence on outcomes of IMV in critically ill patients with ILD. The strength of this review lies in its systematic methodology, including comprehensive literature searches, dual screening and independent risk of bias assessments, ensuring transparency and reproducibility.

However, we anticipate several limitations. The presence of heterogeneous observational studies, with inconsistent reporting of outcomes and ILD subtypes, may cause significant variability. This variability, along with potential biases, will influence the certainty of the findings. Despite this, the review will highlight patterns and variations in outcomes, helping to identify gaps in the literature.

The findings will likely provide insight into current clinical practices and inform future research priorities, including the need for prospective, standardised studies and tailored ventilatory strategies. While definitive conclusions may be limited, the review will guide future investigations and contribute to evidence-based clinical decision-making for this high-risk population.

## Ethics and dissemination

As our study represents a systematic review and meta-analysis of previously published data, ethical approval is not required.

On completion of this systematic review, we believe that the dissemination process will be pivotal to ensure its findings are accessible to the interested actors. This will include its publication in a peer-reviewed, open-access journal and presentation at relevant scientific conferences. Additional dissemination strategies may include engagement with relevant clinical and academic audiences through institutional meetings and professional networks to maximise accessibility and uptake of findings.
