# Supplementary material for: Outcomes of invasive mechanical ventilation in critically ill patients with interstitial lung disease: protocol for a systematic review and meta-analysis
Source: BMJ Open. 2026 Jun 8;16(6):e118165. doi: 10.1136/bmjopen-2026-118165 (PMC13250214; doi:10.1136/bmjopen-2026-118165)
Supplement: Supplementary file 1 [file bmjopen-2026-118165.reviewer_comments.pdf]

PEER REVIEW HISTORY

BMJ Open publishes all reviews undertaken for accepted manuscripts. Reviewers are asked to complete a checklist review form and are provided with free text boxes to elaborate on their assessment. These free text comments are reproduced below.

ARTICLE DETAILS

Title (Provisional)

Outcomes of Invasive Mechanical Ventilation in Critically Ill Patients with Interstitial Lung Disease: protocol for a systematic review and meta-analysis

Authors

Kola, Novis; Parker, Robert; Hall, Angela; spencer, lisa G; Fletcher, Sophie V; Welters, Ingeborg; Johnston, Brian

---

VERSION 1 - REVIEW

**Reviewer**	**1**
**Name**	**Pascoe, Amy**
**Affiliation**	**Monash University**
**Date**	**11-Mar-2026**
**COI**	**None**

Thank you for the opportunity to review this protocol for a systematic review on invasive mechanical ventilation outcomes for critically ill people with ILD. This is an interesting and robust research quesiton, however some additional justification of the planned pooling of ILD variants in the primary analysis should be considered.

Abstract

- please provide proposed search dates in the methods

- please specify what kinds of studies are eligible for inclusion or exclusion

- please provide details on the meta-analysis statistical methods, including if there will be subgroup analyses by ILD variant

Introduction

- lines 95-98 is a single sentence paragraph, I would consider revising.

- please provide citations for the several observational studies mentioned in line 101

- background evidence highlights variation in outcomes across ILD variants so I am still wondering if there will be separate analyses, it has not been made clear at this point in the paper.

Methods

- line 142 refers to study authors and I assume this means the authors of included studies rather than authors of this review, but it is not entirely clear

- how will the authors manage publications in other languages?

- it appears that there is only planned subgroup analyses based on ILD variant if there is significant heteogeneity. Some justification on pooling all variants in the primary analysis given the introduction which emphasised the differences in pathological manifestation and clinical outcomes would be warranted.

Reviewer **2**

Name **Zheng, Jianqiao**

Affiliation **Sichuan University West China Hospital, Department of Anesthesiology**

Date **21-Mar-2026**

COI **None**

Thank you for submitting your manuscript titled "Outcomes of Invasive Mechanical Ventilation in Critically Ill Patients with Interstitial Lung Disease: protocol for a systematic review and meta-analysis" (Manuscript ID: bmjopen-2026-118165) to BMJ OPEN. It was my honor to serve as a reviewer and provide comments on this work.

This protocol is a high-quality, methodologically rigorous, and clinically impactful design for a systematic review and meta-analysis of IMV outcomes in critically ill ILD patients. Minor revisions are recommended, with the following comments:

1 This paper should elaborate on the specific modalities of invasive mechanical ventilation (IMV). For instance, it needs to clarify whether tracheostomy is categorized as a form of IMV.
2 The search terms for literature retrieval should incorporate specific modalities of invasive ventilation to further improve the comprehensiveness of the literature search.
3 It is necessary to clearly state whether grey literature will be included in the study.
4 The definitions of outcome measures remain unclear. For example, terms such as "NIV failure" and "post-discharge respiratory outcomes" have inconsistent definitions in existing literature. The absence of pre-specified unified definitions for such outcomes may introduce heterogeneity into data synthesis and compromise the comparability of pooled results.
5 More detailed data extraction items should be specified, including: (1) basic study information (first author, publication year, study type, sample size); (2) patient characteristics (age, gender, ILD subtype, comorbidities, baseline lung function); (3) intervention details (IMV initiation timing, ventilation parameters, non-invasive ventilation application); (4) outcome measures (specific calculation methods for ventilator-free days, follow-up duration for 1-year mortality).
6 The specific handling methods for missing data should be explicitly stated in the protocol.

7 Although subgroup analyses are planned to explore potential sources of heterogeneity (e.g., ILD subtype, mechanical ventilation type, geographical differences in treatment strategies), the scope of subgroup analyses should be expanded and its dimensions clearly defined. This will facilitate a more comprehensive exploration of heterogeneity sources, such as pulmonary hypertension status, IMV initiation timing, baseline lung function, and geographic region of the study population.

For the assessment of publication bias, a specific threshold for the number of included studies (≥10 studies) should be pre-defined, and funnel plots combined with Egger's test should be used for publication bias assessment when the threshold is met.

VERSION 1 - AUTHOR RESPONSE

Reviewer: 1

Dr. Amy Pascoe, Monash University

Comments to the Author:

Thank you for the opportunity to review this protocol for a systematic review on invasive mechanical ventilation outcomes for critically ill people with ILD. This is an interesting and robust research question, however some additional justification of the planned pooling of ILD variants in the primary analysis should be considered.

Abstract

- please provide proposed search dates in the methods

We have provided these as suggested.

- please specify what kinds of studies are eligible for inclusion or exclusion

We have provided these as suggested.

- please provide details on the meta-analysis statistical methods, including if there will be subgroup analyses by ILD variant

We have provided these as suggested.

Introduction

- lines 95-98 is a single sentence paragraph, I would consider revising.

This has been revised (now line 135-139).

- please provide citations for the several observational studies mentioned in line 101

These have been provided now (Line 144).

- background evidence highlights variation in outcomes across ILD variants so I am still wondering if there will be separate analyses, it has not been made clear at this point in the paper.

Yes, there will be separate analyses based on data availability i.e. if there are enough data to do subgroup analyses.

Methods

- line 142 refers to study authors and I assume this means the authors of included studies rather than authors of this review, but it is not entirely clear

Yes, this has now been edited to make it clear (Line 187).

- how will the authors manage publications in other languages?

This has now been addressed in lines 222-226

- it appears that there is only planned subgroup analyses based on ILD variant if there is significant heteogeneity. Some justification on pooling all variants in the primary analysis given the introduction which emphasised the differences in pathological manifestation and clinical outcomes would be warranted.

We thank the reviewer for this important observation. We acknowledge that ILD comprises a heterogeneous group of conditions with distinct pathological mechanisms and clinical trajectories, which may influence outcomes following invasive mechanical ventilation (IMV).

Our decision to pool all ILD subtypes in the primary analysis is based on the overarching aim of the review, which is to evaluate outcomes of IMV in the broader ILD population as encountered in clinical practice, where management decisions are often made prior to definitive subtype stratification. Pooling allows for a more comprehensive estimate of overall outcomes and improves statistical power, particularly given the anticipated limited sample sizes within individual ILD subtypes.

We recognise, however, that this approach may obscure important subtype-specific differences. To address this, we have planned subgroup analyses by ILD subtype, which will be conducted where sufficient data are available. In addition, we will assess statistical heterogeneity and, where substantial heterogeneity is identified, explore ILD subtype as a potential source.

This has now been reflected on the manuscript (lines 271-281).

Reviewer: 2
Dr. Jianqiao Zheng, Sichuan University West China Hospital
Comments to the Author:
Thank you for submitting your manuscript titled “Outcomes of Invasive Mechanical Ventilation in Critically Ill Patients with Interstitial Lung Disease: protocol for a systematic review and meta-analysis” (Manuscript ID: bmjopen-2026-118165) to BMJ OPEN. It was my honor to serve as a reviewer and provide comments on this work.
This protocol is a high-quality, methodologically rigorous, and clinically impactful design for a systematic review and meta-analysis of IMV outcomes in critically ill ILD patients.

Minor revisions are recommended, with the following comments:
1 This paper should elaborate on the specific modalities of invasive mechanical ventilation (IMV). For instance, it needs to clarify whether tracheostomy is categorized as a form of IMV.

This has been now reflected on the manuscript (line 194-196).

2 The search terms for literature retrieval should incorporate specific modalities of invasive ventilation to further improve the comprehensiveness of the literature search.

We thank the reviewer for the comments. We have reviewed our search strategy with our medical librarian to ensure that specific modalities will be included in our review. We have used IMV as our mesh term and exploded terms under IMV in our search. As such we are confident that studies reporting different modalities of IMV will be included. Where data allow, we intend to report the IMV modalities and undertake subgroup analysis to assess its impact.

3 It is necessary to clearly state whether grey literature will be included in the study.

This has been now reflected on the manuscript (line 251-259).

4 The definitions of outcome measures remain unclear. For example, terms such as "NIV failure" and "post-discharge respiratory outcomes" have inconsistent definitions in existing literature. The absence of pre-specified unified definitions for such outcomes may introduce heterogeneity into data synthesis and compromise the comparability of pooled results.

This has been addressed now (line 208-225).

5 More detailed data extraction items should be specified, including: (1) basic study information (first author, publication year, study type, sample size); (2) patient characteristics (age, gender, ILD subtype, comorbidities, baseline lung function); (3) intervention details (IMV initiation timing, ventilation parameters, non-invasive ventilation application); (4) outcome measures (specific calculation methods for ventilator-free days, follow-up duration for 1-year mortality).

This has been reflected on the manuscript (table 3).

6 The specific handling methods for missing data should be explicitly stated in the protocol.

This has been addressed now on our protocol (line 271-273).

7 Although subgroup analyses are planned to explore potential sources of heterogeneity (e.g., ILD subtype, mechanical ventilation type, geographical differences in treatment strategies), the scope of subgroup analyses should be expanded and its dimensions clearly defined. This will facilitate a more comprehensive exploration of heterogeneity sources, such as pulmonary hypertension status, IMV initiation timing, baseline lung function, and geographic region of the study population.

We appreciate this suggestion and have expanded and clearly defined subgroup analyses (line 329).

8 For the assessment of publication bias, a specific threshold for the number of included studies (≥10 studies) should be pre-defined, and funnel plots combined with Egger's test should be used for publication bias assessment when the threshold is met.

We have clarified the criteria and methods for publication bias assessment (Line 345-347).

VERSION 2 - REVIEW

**Reviewer**	**2**
**Name**	**Zheng, Jianqiao**
**Affiliation**	**Sichuan University West China Hospital, Department of Anesthesiology**
**Date**	**05-May-2026**

COI

This protocol has been carefully revised in accordance with the reviewers' comments. The methodological aspects have been thoroughly improved, and the overall quality of the paper has been further enhanced. We believe that it now meets the standards for publication.
